# The Genetics of Osteosarcoma

**DOI:** 10.1155/2012/627254

**Published:** 2012-05-20

**Authors:** Jeff W. Martin, Jeremy A. Squire, Maria Zielenska

**Affiliations:** ^1^Department of Paediatric Laboratory Medicine, Hospital for Sick Children, Toronto, ON, Canada M5G 1X8; ^2^Department of Pathology and Molecular Medicine, Queen's University, Kingston, ON, Canada K7L 3N6

## Abstract

Osteosarcoma is a primary bone malignancy with a particularly high incidence rate in children and adolescents relative to other age groups. The etiology of this often aggressive cancer is currently unknown, because complicated structural and numeric genomic rearrangements in cancer cells preclude understanding of tumour development. In addition, few consistent genetic changes that may indicate effective molecular therapeutic targets have been reported. However, high-resolution techniques continue to improve knowledge of distinct areas of the genome that are more commonly associated with osteosarcomas. Copy number gains at chromosomes 1p, 1q, 6p, 8q, and 17p as well as copy number losses at chromosomes 3q, 6q, 9, 10, 13, 17p, and 18q have been detected by numerous groups, but definitive oncogenes or tumour suppressor genes remain elusive with respect to many loci. In this paper, we examine studies of the genetics of osteosarcoma to comprehensively describe the heterogeneity and complexity of this cancer.

## 1. Introduction

Osteosarcoma is the most common primary bone malignancy, with a high incidence rate in children and adolescents compared to other age groups. Tumours most often arise in the long bones from osteoid-producing neoplastic cells adjacent to the growth plates, occurring less commonly in the axial skeleton and other nonlong bones [1]. Survival rates for osteosarcoma have remained at 60–70% for localised disease for decades despite ongoing studies [2]. Unlike many sarcomas which are characterised by specific chromosome translocations, complex genomic rearrangements involving any chromosome characterise individual osteosarcoma cells. Because of this few consistent genetic changes that may indicate effective molecular targets for treatment have been reported.

Decades' worth of molecular cytogenetics studies and genomic analyses of osteosarcomas have been completed through karyotyping, comparative genomic hybridisation (CGH), fluorescence *in situ *hybridisation, quantitative PCR, and single-strand conformation polymorphism analysis, among others. Genome-wide association studies utilising single-nucleotide polymorphisms (SNPs) have been used more recently to learn more broadly about osteosarcoma genomics [3]. Resolution of alterations has increased from visualisation at the chromosome level to point mutations, but the genetic etiology of osteosarcoma is still unknown. One consistent finding, however, is the higher incidence of osteosarcoma relative to the general population in individuals with familial Li-Fraumeni syndrome (germline *TP53 *inactivation), hereditary retinoblastoma (germline *RB1 *inactivation), Rothmund-Thomson syndrome (germline *RECQL4 *inactivation), or Bloom or Werner syndrome (germline *BLM *or *WRN *inactivation, resp.) [4–8]. The genes associated with all of these familial syndromes encode protein products necessary to stabilise the genome, and their impairment can manifest in defective maintenance of DNA.

In this paper, we have collected studies of the genetics of osteosarcoma to illustrate the heterogeneity and complexity of this tumour type at the level of the chromosome and gene. Osteosarcoma-specific epigenetic changes, mRNA and protein level aberrations, and changes to microRNA (miRNA) will not be described extensively in this paper. Other publications on these topics exist and offer more thorough descriptions of the epigenetic [9], expression [10, 11], and miRNA profiling [12, 13] of osteosarcoma. To understand the molecular dynamics of this disease at any level, it is important to first recognize the fundamental role of the disruption of cellular mechanisms intended to maintain genomic instability.

## 2. Genomic Instability in Osteosarcoma

Osteosarcoma is characterised by a high level of genomic instability, in particular one subcategory of instability known as chromosomal instability (CIN) [14, 15]. Microsatellite instability (MIN) and CpG island methylator phenotype (CIMP) are two other forms of genomic instability, and they have been described extensively and predominantly in colorectal cancer [16, 17]. CIN is the elevated rate of gain or loss of entire chromosomes or sections of chromosomes [16, 18], and it appears to be significant in the pathogenesis of osteosarcoma tumours, resulting in complicated structural and numerical aberrations and wide variability between cells [19].

CIN is categorised in two subtypes, numerical CIN (N-CIN) and structural CIN (S-CIN). Processes underlying N-CIN are those leading to copy number alterations. N-CIN is manifested in polyploidy, caused by errors in mitosis, aneuploidy, segmental amplifications, or deletions, and unbalanced translocations. S-CIN can result from ineffective DNA damage response mechanisms following exogenous insults or replication errors, leading to aberrant genomic rearrangements, chromosomal breakages, and usually, but not necessarily, gene copy number alterations [20]. Karyotypic complexity in tumours, an end product of CIN, is correlated with higher expression of survival- and tissue invasion-related genes and lower expression of those involved in checking cell cycle regulation and ensuring DNA repair [21].

Mutations or deregulation of genes important for mitotic checkpoints is thought to be the underlying cause of CIN [22]. For example, inactivation of the tumour suppressor proteins p53 and pRB cause CIN *in vivo* [23, 24]. Additionally, mutation of *TP53 *is significantly correlated with high levels of genomic instability in osteosarcoma [25], while mutation of *RB1 *contributes to mitotic missegregation and loss of heterozygosity (LOH) in mice [26]. In a study of 18 osteosarcomas, an association was made between overexpression of *RECQL4*, a gene which encodes a DNA helicase, and S-CIN [27]. Whether mutator mutations are in fact required to induce carcinogenesis by increasing the rate of genetic change is still in question [28].

Telomere maintenance, or lack thereof, is another potential source of the instability typical of osteosarcoma, in addition to reducing the likelihood of favourable outcome in patients with the disease. Telomerase activation is a mechanism by which human cells can bypass their theoretical life span defined by the number of cell divisions required to critically deplete telomere length (the Hayflick limit), thereby avoiding senescence [29]. Rather than activation of the telomerase subunit genes, the alternative lengthening of telomeres' (ALTs) mechanism of preserving telomeres is more frequently observed in sarcomas [30]. Telomerase activation and ALT both contribute to telomere maintenance in osteosarcoma, but ALT seems to be the predominant process [31, 32]. Interestingly, ALT is more common in sarcomas not associated with specific translocations [33] and therefore may be associated with more complex chromosomal aberrations in some tumours [34, 35], including osteosarcomas [36, 37]. In females, shorter telomere length is associated with increased risk of osteosarcoma [38]. Additionally, cellular telomere maintenance is associated with poor outcome for osteosarcoma patients [39], but enzymes facilitating ALT may have potential as therapeutic targets [40].

## 3. Genetic Alterations by Osteosarcoma Subtype

 The vast majority of studies have been descriptions of osteosarcomas focused on the conventional, high-grade subtypes including the chondroblastic, fibroblastic, and osteoblastic variants. These are the most frequently occurring types of osteosarcoma. The rarer subtypes include telangiectatic, small cell, periosteal, high-grade surface, and low-grade osteosarcoma. These forms often present with distinguishing genetic features infrequent in conventional tumours.

### 3.1. Conventional Osteosarcoma

**Figure 1:**
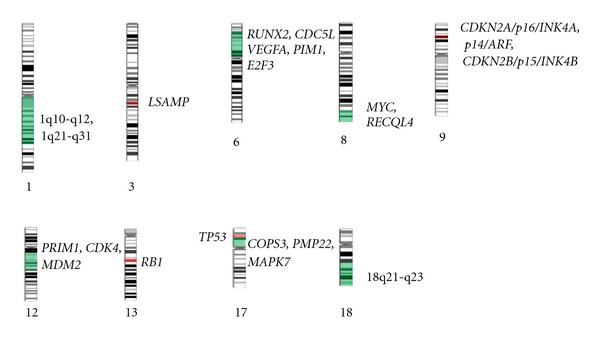
Frequent chromosomal aberrations in sporadic conventional osteosarcoma. Green highlighted areas represent minimal common regions of gain and amplification, or cytobands containing frequently gained and amplified genes. Red highlighted areas represent minimal common regions of loss, or cytobands containing genes frequently lost. Refer to Table 1 and the text for more details regarding minimal common regions and the presence of genetic mutations in some areas of the genome. Chromosome images adapted from the Mitelman Database of Chromosome Aberrations and Gene Fusions in Cancer (http://cgap.nci.nih.gov/Chromosomes/Mitelman/. Accessed January 25, 2012).

 Complex and largely inconsistent genetic alterations are typical of conventional osteosarcoma. Overall, some frequent genetic alterations in conventional osteosarcoma are losses of portions of chromosomes 3q, 6q, 9, 10, 13, 17p, and 18q and gains of portions of chromosomes 1p, 1q, 6p, 8q, and 17p (Table 1; Figure 1). In general, regions in which known tumour suppressor genes are located undergo deletion and mutation events, while those possessing established oncogenes are gained or amplified in cells. Unfortunately, for many of the alterations described in this paper there exist wide ranges of observed frequencies among published reports. These can be due to inconsistencies between materials and methodology used by groups, including differences in the resolution of cytogenetic techniques and platforms, variation between tumour cohorts with respect to staging, histological subtype, and sample size, and whether specimens have been exposed to chemotherapy (chemotherapy drugs may induce DNA damage). The low incidence rate of osteosarcoma exacerbates the limitations on genetic studies of this disease because it lowers the availability of samples. Furthermore, a high level of chromosomal instability is thought to cause the profound intra- and intertumoural heterogeneity observed in and among specimens, in which abnormalities such as heterogeneously staining regions, double-minute chromosomes, and dicentric chromosomes are not uncommon.

Inactivation of *RB1*, located at chromosome 13q14.2, is frequent in sporadic osteosarcoma, and when it occurs due to germline mutation, osteosarcoma incidence significantly increases [41]. *RB1 *encodes the tumour suppressor protein pRB which is essential in preventing cell cycle progression through G1/S following DNA damage. Mechanistically, the protein inhibits members of the E2F transcription factor family, a process that requires strict regulation of the cyclins, cyclin-dependent kinases (CDKs), and cyclin-dependent kinase inhibitors (CDKNs), to promote stability of the genome [42]. LOH or deletion of the *RB1 *locus has been detected in 19–67% of tumours [43–55], and *RB1 *mutations have been detected in about 25–35% of cases [56]. Either type of alteration is associated with inactivation of *RB1 *expression in about 50% of tumours [46].

 Loss of cellular control of other components of the pRB pathway is often deduced upon observing genetic alterations in osteosarcoma tumours. As such, pRB-independent mechanisms of pRB pathway deregulation may be present in addition to pRB inactivation. Amplification of the cyclin-dependent kinase gene *CDK4 *(chromosome 12q13-14) has been detected in approximately 10% of tumours [45, 57]. Approximately 41% of tumours possess amplification of the DNA primase gene *PRIM1*, which is also at chromosome 12q13 [58]. Both PRIM1 and CDK4 are involved in different aspects of the cell cycle phase transition from G1 to S, but the consequences of increased copy number of both genes are unknown. On the other hand, genomic losses of the CDKN genes, all of which encode tumour suppressor proteins that inactivate the CDK proteins, are also frequent. The genes *CDKN2A/p16/INK4A*, *p14/ARF*, and *CDKN2B/p15/INK4B* are located at chromosome 9p21, and *CDKN2A/p16 *alteration has been implicated in osteosarcoma development [59]. Chromosome 9p21 undergoes deletion in 5–21% of osteosarcomas [48, 60–63].

Deregulation of *TP53 *is also thought to be significant in the development of osteosarcoma and occurs due to mutations of the gene or gross changes to the gene locus at 17p13.1. Like pRB, the p53 protein is a tumour suppressor that is activated upon DNA damage recognition and can induce cellular quiescence, senescence, or apoptosis. However, p53 is by far the more commonly inactivated protein in human cancer [64]. Individuals with the Li-Fraumeni syndrome, the manifestation of germline *TP53 *mutations, have an increased incidence of osteosarcoma [4, 6]. LOH and deletions of the 17p13.1 locus have been detected in 29–42% of sporadic osteosarcomas [44, 62, 65, 66]. Mutations of *TP53* are present in 10–39% of cases [25, 44, 47, 48, 56, 62, 67–71].

Direct inactivation of *TP53 *expression is only one mechanism by which the p53 pathway can be disrupted. Functional inactivation of p53 at the posttranslational level can also occur through regulation by tumourigenic proteins. The oncoprotein MDM2 is a well-described inhibitor of p53, functioning both in the promotion of p53 degradation and the downregulation of its transcription. Amplification of *MDM2 *(chromosome 12q15) is a relatively infrequent event in primary osteosarcoma, occurring in 3–25% of tumours [47, 48, 57, 72, 73] but appears to be considerably more frequent in metastases and recurrences [47, 73, 74]. Nearby the *TP53 *locus at chromosome 17p11.2-p12 is another focus of amplification that leads to increased copy number of *COPS3, PMP22, *and *MAPK7*, among other genes. Amplification of chromosome 17p11.2-p12 is more frequent than that of chromosome 12q15, at a range of 20% to 78% of tumours [20, 49, 52, 55, 65, 68, 70, 75–80]. *COPS3* is strongly suspected to be the amplicon target because, like *MDM2*, it has an important role in promoting proteasome-mediated degradation of p53.

Instability of chromosome 8q has been described by many laboratories, with *MYC *(cytoband 8q24.21, also known as *c-MYC*) being gained at varying frequencies. An early report sets the frequency of amplification at 7%, and those events only occurred in tumours from adult patients [81]. Other groups have reported frequencies of gain and amplification of *MYC *at 14–67% [20, 45, 49, 55, 71, 78, 82, 83]. However, other regions of 8q, including 8q23-qter, 8q21.3-8q23, and 8q21 commonly undergo copy number increases as well [20, 49, 55, 77–79, 83, 84], suggesting that other oncogenes located within these bands could have roles in osteosarcoma pathogenesis [82].

Aberrations of the *RECQL4 *gene (8q24.4) are also associated with osteosarcoma development. Loss of RECQL4 function via truncating mutation in individuals with the autosomal recessive familial Rothmund-Thomson syndrome results in significantly higher risk of osteosarcoma [7], but in sporadic osteosarcoma the rate of *RECQL4 *mutation is less than 5% [85]. However, increased copy number and increased protein expression of *RECQL4 *have been reported as a frequent event in sporadic osteosarcoma [27]. Bloom syndrome and Werner syndrome are two additional autosomal recessive syndromes that predispose affected individuals to osteosarcoma [86, 87]. Both syndromes result from genomic instability caused by hereditary mutation of a *RECQL *family DNA helicase gene [8]: Bloom syndrome due to mutation of *BLM *(*RECQL3*) located at chromosome 15q26.1 and Werner syndrome due to mutation of *WRN *(*RECQL2*) located at chromosome 8p12. Distinct regions of chromosome arms 15q and 8p are prone to inconsistent rearrangements and copy number alterations in sporadic osteosarcoma, frequently as amplicons within 15q and loss of 8p regions [50, 75, 78, 88].

Amplifications within the short arm of chromosome 6, with a minimal common region at 6p12-p21, have been frequently observed at rates of 16–75% in conventional osteosarcoma tumour specimens [9, 20, 45, 49, 65, 77, 78, 82, 89–92], including those from biopsy, surgical resection, and metastases [89]. Data obtained using 10 osteosarcoma patient samples indicate amplification-related overexpression of genes within the 6p12-p21 region [93]. Notably, in addition to this, a conditional mouse model of osteosarcoma demonstrated overexpression of genes within mouse genomic regions homologous to human 6p12-p21 [94], consistent with observations of 6p deregulation in human osteosarcoma.

A number of genes with oncogenic potential lie within chromosome 6p12-p21 and in close proximity to this region. *E2F3* (6p22.3) is gained or amplified in approximately 60% of osteosarcomas [92] and encodes the E2F3 transcription factor. An increased level of E2F3 is associated with the accumulation of DNA damage [95] and increased proliferation rate in cancer [96, 97]. *PIM1* is a protooncogene located at 6p21.2 that encodes a serine/threonine-protein kinase and whose overexpression is associated with high-grade prostate cancer [98]. *VEGFA *(6p21.1) is amplified in 25% of a cohort of osteosarcoma specimens [99], and its protein product promotes angiogenesis and blood vessel permeability in cancer [100]. Also at cytoband 6p21.1 is the human cyclin D3 gene *CCND3*, which is commonly amplified in other cancers [101, 102], *CDC5L *(*cell division cycle 5-like*), and *RUNX2 *(*runt-related transcription factor 2*). *CDC5L *encodes a cell cycle regulator which may function in human osteosarcoma [89], and its overexpression may promote mitotic entry and shorten the G2 phase [103]. *RUNX2 *encodes a transcription factor important in osteogenesis [104] and has been expressed in up to 87% of tumour specimens, including biopsy samples, implying that alteration of 6p12-p21 may be an early event in the disease [45, 89]. In another report, gain-related overexpression of *RUNX2* was observed in 60% of the analysed osteosarcoma tumours [9], and overexpression of *RUNX2 *is correlated with poor response to chemotherapy [93].

Other genomic regions frequently altered in copy number but whose potential gene targets are less well characterised in osteosarcoma have been abundantly described. Amplifications of chromosome 1q, at minimal regions including 1q10-q12 and 1q21-q31, occur in 6–59% of tumours in addition to other rearrangements of 1q [50, 55, 77, 82–84]. Portions of chromosome 17q undergo mixed duplication and deletion events in osteosarcoma [65, 75], and LOH of the tumour suppressor gene *BRCA1 *(17q21.31) has been detected [66].

Loss of chromosome 3q, with a minimal common region at 3q13.31, has been observed in 6–80% of tumours [44, 45, 49–55]. The presence of a novel gene, *limbic system-associated membrane protein* (*LSAMP*), at 3q13.31 is suggested to have a significant tumour suppressive role in osteosarcoma [49]. Another group has suggested the presence of an osteosarcoma tumour suppressor gene at locus 3q26.2-q26.3 based on findings of frequent LOH of this region [105].

LOH at chromosome 10q26 has been reported in 60% of a cohort, and the genes *BUB3 *and *fibroblast growth factor receptor 2 *(*FGFR2*) are suspected to be of importance in this region. *BUB3 *encodes a mitotic checkpoint protein and could have a role in maintaining genomic stability, while *FGFR2 *is involved in skeletal formation [106]. Deletion of *WWOX *(chromosome 16q23.1-q23.2) has been reported in 30% of osteosarcomas [107], but, perhaps more importantly, reduction of its expression occurs in up to 58% of specimens and is associated with elevated *RUNX2 *expression [108]. In a study of 91 osteosarcomas, LOH of the tumour-suppressor gene *APC *(chromosome 5q21) was detected in 62% of cases, while both amplification and deletion (the latter the more frequent event) of *TWIST *(chromosome 7p21) and *MET *(chromosome 7q31) were detected [109] (Table 1).

As at chromosome 3q, LOH at chromosome 18q has been frequently observed, but no studies have defined a distinct tumour suppressor gene important in osteosarcoma. Chromosome 18q is lost in 31–64% of specimens [44, 53, 110]. This portion of chromosome 18 contains a locus of susceptibility near 18q21-q23 that is linked to the Paget disease of bone (PDB) [111, 112]. PDB is a disorder of older adults which leads to osteosarcoma in about 1% of pagetic patients, particularly in the case of familial PDB [113]. A minimal common region of loss in osteosarcoma has been identified as overlapping the locus associated with PDB [110, 114]. This region excludes previously identified candidate genes including *TNFRSF11A*, which encodes RANK [114], and *deleted in colorectal cancer *(*DCC*), which nonetheless is frequently reduced in expression in osteosarcoma [115].

### 3.2. Telangiectatic Osteosarcoma

 Telangiectatic osteosarcoma is a rare subtype of the disease, accounting for between 2 and 12% of cases [116]. Few cytogenetic studies of this subtype have been published, and most of the published observations have described individual cases. Tumour cells from two female patients with telangiectatic osteosarcoma were predominantly normal genomically (46xx), though one tumour possessed cells with trisomy 3 and the other possessed pseudotetraploidy and telomeric associations in a few cells [117]. One group has reported a *TP53 *mutation in a single case which otherwise had normal *RB1 *and no copy number change in *MDM2* [56]. Other studies have reported a constitutional inversion at chromosome 9p11-9q12 in a patient, along with non-clonal balanced translocations in the tumour [20], and a familial occurrence of telangiectatic osteosarcoma in cousins, but without any apparent hereditary components [118]. Gains of chromosomes 6p12-p21, 8q, 12q13-q15, and 14q, along with loss of 2q24-qter, have been observed in one tumour [50]. Overall, however, reported cases of telangiectatic osteosarcoma appear to have relatively few structural and numeric chromosomal alterations in comparison to the other subtypes of the disease [50, 91].

### 3.3. Small Cell Osteosarcoma

 Histologically, small cell osteosarcoma can be mistaken for Ewing's sarcoma, but cytogenetically they lack any consistent genetic alteration. The *t*(11; 22)(q24; q12) translocation, typical of Ewing's sarcoma, has been reported in one case of small cell osteosarcoma tumour [119]. These results have not been replicated in subsequent studies [120, 121], but a *EWSR1-CREB3L1* fusion transcript was detected in a small cell osteosarcoma tumour [122]. Complex structural and numerical rearrangements of multiple chromosomes have been found in two cases of this subtype studied by different labs [75, 88], one of which possessed amplification of 6p12-p21. A study of *MDM2 *copy number and *TP53 *and* RB1 *mutations in a single small cell osteosarcoma specimen reported normal *TP53*, *RB1, *and *MDM2* [56]. Another study found complex structural rearrangements of chromosomes 6, 16, and 17 and monoallelic deletion of *TP53* in one tumour [123].

### 3.4. Periosteal Osteosarcoma

 The genetic alterations observed in this subtype have been largely inconsistent. Cells in one case were found only with an additional copy of chromosome 17 [117], in another possessed only gain of 20q12-q13.2 [50], while in a third case were the only cells in a cohort of 31 osteosarcomas of various subtypes to have no DNA copy number aberrations at all [83]. Another study of three periosteal osteosarcomas reported gains of 2q, 5p, 8q, portions of 12p and 12q, and chromosomes 14 and 21, as well as losses of chromosomes 6, 8p, and 13. The same study reported focal amplifications of 8q11-q24 in one case and of 12q11-q15 in each of the other two cases, in addition to various other amplicons [75]. Complex chromosomal alterations have been reported by others [124, 125], and point mutations in *TP53 *have also been detected [126].

### 3.5. High-Grade Surface Osteosarcoma

 Amplification of the *sarcoma amplified sequence *(*SAS*) gene (located at 12q14.3-15) was reported in a single case of high-grade surface osteosarcoma and six cases of low-grade surface tumours [127]. However, there are no published observations of cytogenetic alterations in prechemotherapy biopsies of high-grade surface osteosarcomas.

### 3.6. Low-Grade Osteosarcoma

 In one CGH study of low-grade central osteosarcoma, six of seven specimens possessed a single copy number change and there were recurrent gains at 12q13-q14, 12p, and 6p21.1-p21.3 among the cases [128]. Amplification of oncogene *ERBB2 *(chromosome 17q12) has been detected in 26% of low-grade tumours [129]. Other researchers assayed 21 tumours of this subtype by sequencing for *TP53 *and the oncogene *HRAS*, and no specimens possessed mutations of either gene. However, amplification of *MDM2 *was detected in 19% of the 21 cases [130]. A separate study described amplification of chromosome 12q13-q15 in five low-grade central osteosarcomas and amplification-related overexpression of *MDM2 *and *CDK4* which lie within the region [131]. Both the overall lack of complex chromosomal aberrations and the low frequency of *TP53 *mutations differentiate this subtype from conventional high-grade osteosarcoma.

 Parosteal osteosarcoma is characterised by a high rate of *MDM2 *amplification (chromosome 12q13-q14), in up to 83% of studied tumours [72, 132]. Chromosome 12q13-q15 amplification products have also been found within supernumerary ring chromosomes in another study that detected amplification of the region in 100% of the specimens examined [133].

## 4. Conclusions

**Figure 2:**
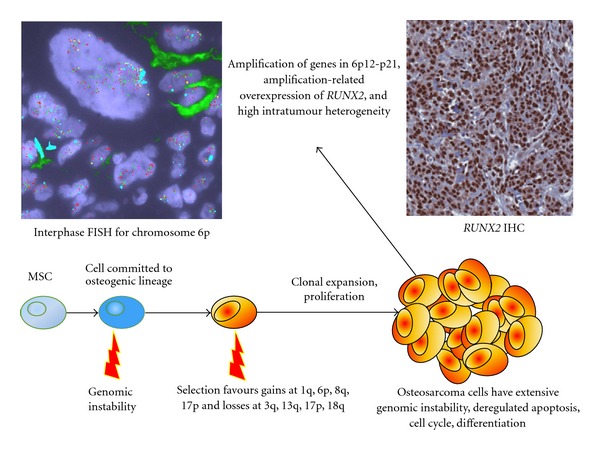
Chromosome 6p rearrangement. Chromosome 6p12-p21, which contains *RUNX2*, frequently undergoes complex rearrangements in osteosarcoma and is one example of a distinct genomic locus that undergoes such alterations in this cancer. In this case, there is gain and amplification of the labeled genes (with wide variation between cells) as shown in the image of interphase fluorescence *in situ *hybridisation (FISH) for chromosome 6p. The FISH experiment employed probes for *FBXO9* (yellow), *RUNX2* (orange), *PIM1* (green), *E2F3* (red), and the centromere of chromosome 6 (light blue). The RUNX2 immunohistochemistry (IHC) image was obtained after staining for RUNX2 protein. High levels of the protein were nearly ubiquitous in the nuclei of cells and were associated with genetic amplification of *RUNX2*. The FISH and IHC images were obtained via experiments performed on serial formalin-fixed paraffin-embedded sections of one osteoblastic (conventional) osteosarcoma tissue specimen. MSC, mesenchymal stem cell.

 Osteosarcoma is characterised by extensive and heterogeneous genetic complexity, which is reflected in the similarly complex epigenetic and expression alterations in tumours [134] and is visually apparent in the results of quantitative research (Figure 2). Mechanisms of genomic instability may be facilitated by the repetitive DNA sequences ubiquitous in the human genome, particularly low copy repeats [92, 135], but this area still requires further study. Unfortunately, even though several alterations are relatively consistent across cohorts of tumours, the accumulated knowledge of genetic changes in osteosarcoma has yet to significantly impact survival rates. Clinical markers continue to be the most reliable indicators for prognostication [136]. Overall, the multitude of genetics studies of osteosarcoma serves to illustrate the extremes to which DNA alterations in cancer can reach, but it is hoped that accurate biomarkers and targeted therapies will soon be revealed for this disease.

## Figures and Tables

**Table 1:** Frequent genetic alterations in sporadic conventional osteosarcoma.

Genomic region	Event	Frequency	Effected genes		References
			Tumour suppressor gene(s)	Oncogene(s)	
1q10-q12, 1q21-q31	Amp	6–59%			[50, 55, 77, 82–84]
3q13.31	Del, LOH	6–80%	*LSAMP*		[44, 45, 49–55]
5q21	LOH	62%	*APC*		[109]
6p12-p21	Gain, Amp	16–75%		*RUNX2, CDC5L, VEGFA, PIM1*	[9, 20, 45, 49, 65, 77, 78, 82, 89–92]
6p22.3	Gain, Amp	60%		*E2F3*	[92]
7p21 7q31	Del Amp Del Amp	36% 14% 41% 9%		*TWIST* * MET *	[109]
8q24.21	Amp	7–67%		*MYC*	[20, 45, 49, 55, 71, 78, 81–83]
8q24.4	Mut	<5%	*RECQL4*		[85]
	Gain	33%	*RECQL4*		[27]
9p21	Del	5–21%	*p16/INK4A,* * p14/ARF,* * p15/INK4B*		[48, 60–63]
10q26	LOH	60%	*BUB3,* * FGFR2*		[106]
12q13	Amp	41%		*PRIM1*	[58]
12q14	Amp	10%		*CDK4*	[45, 57]
12q15	Amp	3–25%		*MDM2*	[47, 48, 57, 72, 73]
13q14.2	LOH	19–67%	*RB1*		[43–55]
	Mut	25–35%	*RB1*		[46, 56]
16q23.1-q23.2	Del	30%	*WWOX*		[107]
17p11.2-p12	Amp	20–78%		*COPS3, PMP22, MAPK7*	[20, 49, 52, 55, 65, 68, 70, 75–80]
17p13.1	Del, LOH	29–42%	*TP53*		[44, 62, 65]
	Mut	10–39%	*TP53*		[25, 44, 47, 48, 56, 62, 67–71]
18q (MCR 18q21-q23)	Del	31–64%			[44, 53, 110, 114]

MCR, minimal common region; Del, deletion; Amp, amplification; Mut, mutation.
